# Disentangling Unusual Catalytic Properties and the Role of the [4Fe-4S] Cluster of Three Endonuclease III from the Extremophile *D. radiodurans*

**DOI:** 10.3390/molecules27134270

**Published:** 2022-07-02

**Authors:** Filipe Rollo, Patricia T. Borges, Célia M. Silveira, Margarida T. G. Rosa, Smilja Todorovic, Elin Moe

**Affiliations:** Instituto de Tecnologia Química e Biológica António Xavier, Universidade NOVA de Lisboa, Av. da República, 2780-157 Oeiras, Portugal; filipe.rollo@itqb.unl.pt (F.R.); pborges@itqb.unl.pt (P.T.B.); celiasilveira@itqb.unl.pt (C.M.S.); amgomesrosa@gmail.com (M.T.G.R.); smilja@itqb.unl.pt (S.T.)

**Keywords:** DNA repair, Base Excision Repair, DNA glycosylase, mutants, spectroscopy

## Abstract

Endonuclease III (EndoIII) is a bifunctional DNA glycosylase with specificity for a broad range of oxidized DNA lesions. The genome of an extremely radiation- and desiccation-resistant bacterium, *Deinococcus radiodurans*, possesses three genes encoding for EndoIII-like enzymes (DrEndoIII1, DrEndoIII2 and DrEndoIII3), which reveal different types of catalytic activities. DrEndoIII2 acts as the main EndoIII in this organism, while DrEndoIII1 and 3 demonstrate unusual and no EndoIII activity, respectively. In order to understand the role of DrEndoIII1 and DrEndoIII3 in *D. radiodurans*, we have generated mutants which target non-conserved residues in positions considered essential for classic EndoIII activity. In parallel, we have substituted residues coordinating the iron atoms in the [4Fe-4S] cluster in DrEndoIII2, aiming at elucidating the role of the cluster in these enzymes. Our results demonstrate that the amino acid substitutions in DrEndoIII1 reduce the enzyme activity without altering the overall structure, revealing that the residues found in the wild-type enzyme are essential for its unusual activity. The attempt to generate catalytic activity of DrEndoIII3 by re-designing its catalytic pocket was unsuccessful. A mutation of the iron-coordinating cysteine 199 in DrEndoIII2 appears to compromise the structural integrity and induce the formation of a [3Fe-4S] cluster, but apparently without affecting the activity. Taken together, we provide important structural and mechanistic insights into the three EndoIIIs, which will help us disentangle the open questions related to their presence in *D. radiodurans* and their particularities.

## 1. Introduction

Endonuclease III (EndoIII) is a ubiquitous bifunctional DNA glycosylase belonging to the helix-hairpin-helix (HhH)-GDP superfamily of DNA glycosylases. It is active for a broad range of oxidized pyrimidine lesions, removing numerous forms of modified thymine and cytosine bases from DNA, such as thymine glycol (Tg), a major stable oxidative modification of thymine, via the Base Excision Repair (BER) pathway [1]. The EndoIII enzymes are highly conserved across all kingdoms of life and are thus very important for genome maintenance from bacteria to man. It was recently shown that the gene encoding for the human EndoIII homologue, hNTH1, is associated with development of adenomatous polyposis and colorectal cancer [2].

The crystal structures of EndoIIIs from *E. coli* (EcEndoIII) [3] *D. radiodurans* (DrEndoIII1 and DrEndoIII3) [4] and *G. stearothermophilus* (GsEndoIII) [5] revealed that these enzymes comprise two domains divided by a positively charged DNA binding cleft containing the HhH motif and the catalytic residues Asp and Lys. Domain A consists of 4 alpha-helices, the N and C termini, a [4Fe-4S] cluster loop (FCL) and a [4Fe-4S] cluster; the domain B consist of six alpha-helices and two DNA intercalating loops (DIL). Recently the crystal structure of the catalytic domain of human EndoIII (hNTH1) was also determined, revealing the same organization (A and B) as its bacterial homologues [6]. Thus, results from studies of bacterial EndoIII enzymes may also be relevant for understanding the molecular mechanisms underlying the function of the human enzyme.

*Deinococcus radiodurans* is an extremely radiation- and desiccation-resistant gram-positive bacterium, which can withstand 200 times higher doses of ionizing irradiation than other bacteria without losing viability [7,8,9,10]. The mechanism of *D. radiodurans* resistance is not known, but a combination of factors, such as a condensed and high genome copy number (between four and ten copies during the exponential phase of growth), proteome protection against reactive oxygen species (ROS), high number of DNA glycosylases and efficient DNA-repair machineries [9,11] are designated as possible key players in its innate resistance to DNA damage.

*D. radiodurans* possesses three genes encoding EndoIII-like enzymes (DrEndoIII1, DrEndoIII2 and DrEndoIII3), while other bacteria typically possess one. The insights obtained from the crystal structures of DrEndoIII1 and DrEndoIII3 and the homology model for DrEndoIII2, revealed that the three DrEndoIIIs share a similar fold among themselves and with that of EcEndoIII and GsEndoIII [3,4,5,12]. Only DrEndoIII1 and DrEndoIII2 possess activity towards common EndoIII substrates (e.g., Tg, 5-hydroxycytosine (5OHC)).

DrEndoIII2 is highly efficient and possesses broad substrate specificity, with catalytic properties that surpass those of EcEndoIII and Human Endonuclease III (hNTH1) [12]. DrEndoIII1 displays a lower activity towards the common substrates and act mostly as a monofunctional DNA glycosylase. It was also found to be capable of processing single-stranded DNA (ssDNA) substrates [12]. Previous comparative structural analysis suggested that amino acid substitutions in two DNA interacting loops (DIL1 and DIL2) and in the FeS cluster loop (a structurally conserved DNA binding motif) could be the origin of the reduced activity of DrEndoIII1 [4]. DIL 1 and DIL 2 are involved in the stabilization of DNA when the damaged base is flipped out of the DNA and into the active site pocket. Two of the main residues involved in this stabilization are Gln53 and Leu93 in DrEndoIII2. In DrEndoIII1 these residues are substituted by Arg61 and Tyr100 [4].

Despite its preserved fold and conserved catalytic residues (Lys and Asp) DrEndoIII3 shows no detectable enzymatic activity towards common EndoIII substrates at standard assay conditions, however it possesses the highest affinity for damaged DNA [4,12]. Structural analysis of DrEndoIII3 revealed a π-stacking interaction between two arginines (Arg139 and Arg298) at the entrance of the catalytic site, which may prevent the substrate from fully entering the binding pocket for processing by the enzyme [12]. It was also suggested that the lack of activity could be caused by substitutions of conserved Thr and His close to the catalytic Asp (Gly250 and Asn251) in DrEndoIII3. These residues are proposed to be important for stabilization of the phosphate backbone of the DNA upon flipping out the damaged base into the catalytic pocket [12].

The unique property of the HhH-GDP family is the presence of FeS cluster loop and a [4Fe-4S] cluster coordinated by four cysteines [3,13], the role of which is still unclear. It was initially proposed that it has either a structural or a regulatory role [13,14]. Later it was suggested that it is involved in DNA-mediated signaling, shuttling between +2 and +3 redox states [15] upon binding to DNA. Moe et al. more recently revealed that the cluster in DrEndoIII2 becomes redox active not only upon binding to DNA but also to other negatively charged entities [16]. The cluster was proposed to undergo +2 to +1 redox transition, as in the case of 4Fe-S clusters in ferredoxins [15,16,17].

Here we have generated variants of each of the studied DrEndoIII in order to address their distinct and specific features. Mutants of DrEndoIII1 and DrEndoIII3 were constructed in order to address its poor/absent EndoIII activity. In both cases, atypical residues in key positions for classic EndoIII activity were targeted and substituted with conserved residues found in other bacterial EndoIIIs. In DrEndoIII1 we substituted Arg61 and Tyr100 in DIL1 and DIL2 with the equivalent residues found in DrEndoIII2 (Glu and Leu). In the case of DrEndoIII3, we substituted Gly250 and Asn251 with Thr and His close to the catalytic Asp as in EndoIII2 and Arg139 with Asp, in order to disrupt the π-stacking interaction at the entrance of the catalytic pocket. In the well-characterized EndoIII2, we have probed the role of the [4Fe-4S] cluster, by substituting the Fe atom coordinating cysteines. We provide here a thorough characterization of the structural and functional properties of the obtained mutants, using a toolbox of crystallographic, biochemical, spectroscopic, and electrochemical approaches.

## 2. Results and Discussion

### 2.1. Analysis of DrEndoIII1 and DrEndoIII3 Mutants

#### 2.1.1. Protein Expression

The DrEndoIII1, DrEndoIII2 and DrEndoIII3 enzymes and their variants DrEndoIII1 R61Q, DrEndoIII1 Y100L, DrEndoIII3 G250T/N251H were expressed and purified as stable monomeric proteins. On average, the native enzymes were produced with two-times higher yield than the mutant enzymes (20 mg/L and 8–10 mg/L respectively). A very low yield (0.6 mg/L) was obtained for DrEndoIII3 R139D mutant.

#### 2.1.2. Biochemical Characterization

**Figure 1:**
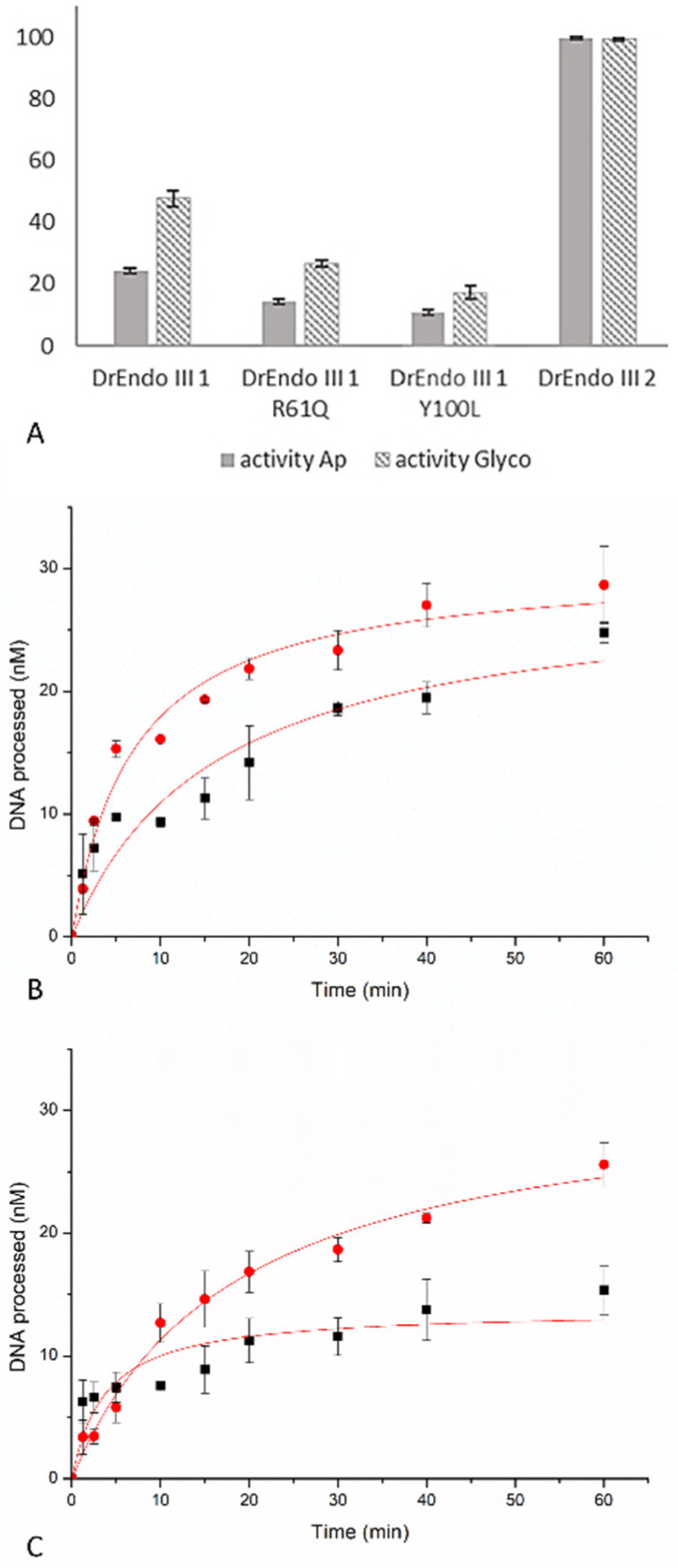
Processing of thymine glycol (Tg) (plain bars) and Ab-site (striped bars), in percentage, by DrEndoIII1 wild-type enzyme and its respective mutants compared to DrEndoIII2 activity. Histograms represent means and standard deviations of at least three independent experiments (**A**). Time course experiments of Tg-DNA processing by DrEndoIII1 R61Q (**B**) and DrEndoIII1 Y100L (**C**). For each experiment, both the DNA glycosylase (red line) and the bifunctional activities (black line) were measured.

In order to evaluate the effect of substitution of the atypical residues in DrEndoIII1 and DrEndoIII3, biochemical assays were conducted. DNA glycosylase and AP-lyase (DNA incision) activity measurements were performed on a 5′-FAM labeled 35mer DNA oligonucleotide containing the common EndoIII substrates, Tg and an abasic site, both opposite of a G at a constant concentration of 75 nM and 75 nM protein. We observe that both DrEndoIII1 mutants possess bifunctional and DNA glycosylase activity, which is, however, reduced compared to DrEndoIII1 and DrEndoIII2 (Figure 1A). Neither DrEndoIII3 G250T/N251H nor DrEndoIII3 R139D demonstrated any activity towards these substrates (Figure S1A–C).

It can be further observed that the activity of DrEndoIII1 Y100L was more affected than that of DrEndoIII1 R61Q. The latter revealed a decrease of approximately 42% and 45% for AP-lyase and DNA glycosylase activities, respectively, whereas Y100L showed a decrease of 55% and 64% when compared with the native enzyme.

To further investigate the enzymatic activity and behavior of the DrEndoIII1 mutants, time course activity measurements were conducted. Progress curves were generated with Tg:G DNA at 75 nM (the same concentration used in endpoint activity assays) and 750 nM of each enzyme. Both glycosylase and AP-lyase activities were measured. The obtained results are in accordance with our previous endpoint analysis, confirming that the activity of the DrEndoIII1 mutants was severely affected by the amino acid substitutions, despite the use of 10x higher concentration of the enzyme than of the substrate. Compared to previous analysis of wild-type DrEndoIII1, which results in full product formation using the same enzyme and substrate concentration [12], DrEndoIII1 R61Q processed approximately one-third (25 nM) of the substrate compared to the native enzyme (Figure 1B). DrEndoIII1 Y100L shows more compromised activity when compared with wild-type and DrEndoIII1 R61Q (Figure 1C).

**Figure 2:**
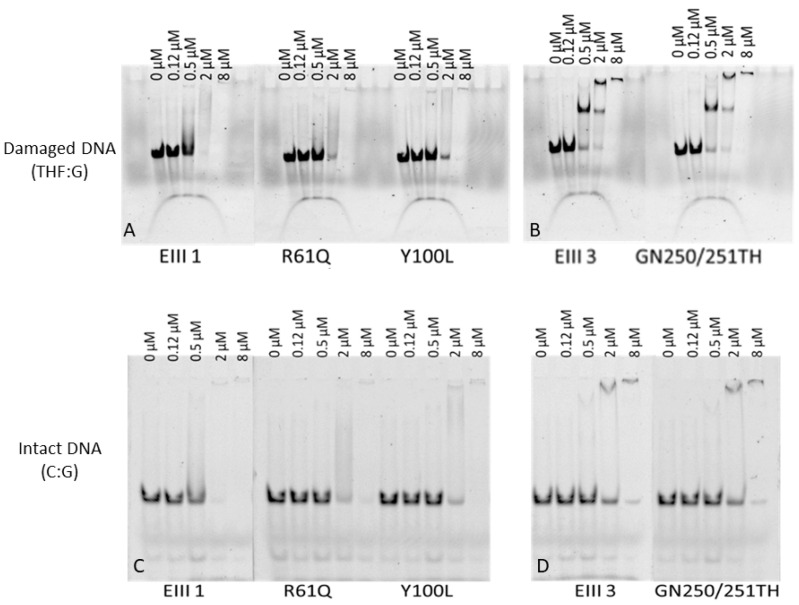
Electrophoretic mobility shift assays (EMSA) of *D. radiodurans* DrEndoIII1, DrEndoIII1 R61Q, DrEndoIII1 Y100L, DrEndoIII3 and DrEndoIII3 GN250/251TH binding to 5′-FAM labeled 35mer dsDNA containing either damaged DNA THF:G (**A**,**B**) and an intact DNA C:G (**C**,**D**).

In order to identify molecular factors underlying the reduced activity of the mutants, the effect of the amino acid substitutions on the substrate binding was analyzed by Electrophoretic mobility shift assays (EMSA). Our results demonstrate that the affinity of the DrEndoIII1 mutants for the substrates (with THF:G mispair and C:G pair) is reduced when compared with the wild-type enzyme (Figure 2).

While native DrEndoIII1 needs 0.5 µM of enzyme to interact with the substrate, the mutants require more than 2 µM of enzyme to form complex with either damaged or undamaged substrate DNA substrate. For DrEndoIII3 GN250/251TH the EMSA results indicates that the GN to TH substitutions had no effect on the enzyme affinity (Figure 2B,D). The EMSA assays also suggest that at higher enzyme concentration, DrEndoIII3 forms a higher oligomeric form binding to the THF-containing substrate (Figure 2B) but not to the intact DNA C:G (Figure 2D).

Taken together we demonstrate here that the substitutions of Arg61 and Tyr100 to Gln and Leu result in a reduced activity of the mutants compared to the wild-type enzyme, thus indicating that the original residues are important for the specific function of the protein in *D. radiodurans*. The decreased activity is in both cases caused by a lower substrate affinity. In the case of the R61Q mutant, this probably originates from the substitution of a positively charged residue with a neutral one. In the Y100L mutant it may be caused by a reduced steric fit of the Gln to the opposite strand of the flipped-out base compared to Tyr. MutY also possesses a Tyr residue in the same position as DrEndoIII1 [5,18], thus it is possible that this residue in some HhH DNA glycosylases is optimized for stabilization of the substrate upon DNA binding.

Our results furthermore demonstrated that neither the R139D nor the GN to TH substitution in DrEndoIII3 led to an induction of endonuclease-type activity of DrEndoIII3 on the tested substrates. Although no catalytic alterations were observed for the DrEndoIII3 mutants, the substrate binding analysis confirmed the previously observed stronger substrate affinity of this enzyme to damaged DNA compared to DrEndoIII1 [12]. A formation of higher oligomeric forms of both DrEndoIII3 and the GN to TH mutant in the presence of damaged DNA was also observed. This had previously been shown for DrEndoIII1 [12], however, this was not visible in the experiments with DrEndoIII1 performed here. In the previous DNA binding studies, a 16 bp THF substrate was used, while in the present study the DNA is larger (45 bp), which could explain the difference observed. Dimer formation has also previously been observed for hNTH1 and was suggested to be involved in AP-lyase regulation mechanisms [19]. The observation of an oligomeric form of DrEndoIII3, at high enzyme concentration, is thus important as it strengthens the previous suggestion that this enzyme may play an alternative role in BER in *D. radiodurans* [12].

#### 2.1.3. Crystal Structure Determination

In order to analyze eventual structural changes imposed by the amino acid substitutions, we determined the crystal structures of the mutants. Brown/orange-colored crystals of DrEndoIII1 R61Q and Y100L and DrEndoIII3 G250T/N251H were obtained and used for data collection on the BL13-XALOC beamline at the ALBA synchrotron. The asymmetric units of the crystals included one monomer as in solution.

The crystal structures were determined and refined to resolutions of 1.38, 1.95 and 1.89 Å for DrEndoIII1 R61Q, DrEndoIII1 Y100L and DrEndoIII3 G250T/N251H, respectively (Table S2). The polypeptide chain of each mutant could be traced in the electron density within residues ranging from amino acid 19–255 for DrEndoIII1 R61Q and Y100L, and 87–324 for the DrEndoIII3 G250T/N251H. The residues 1–18 of DrEndoIII1 mutants and the 1–86 in DrEndoIII3 double mutant, corresponding to the N-terminal, were not visible in the electron density. This was also the case for the C-terminal residues 255–259 in DrEndoIII1 mutants and 324–338 in DrEndoIII3 double mutant.

The electron density in all the mutants’ crystal structures enabled the localization of a [4Fe-4S] iron-sulfur cluster, containing four iron and four sulfide ions. Additionally, the DrEndoIII1 R61Q and Y100L crystal structures include two magnesium ions, and the DrEndoIII3 G250T/N251H crystal structure has two chloride ions. Most of the residues of these structures are within the most favored region of the Ramachandran plot (Table S2).

**Figure 3:**
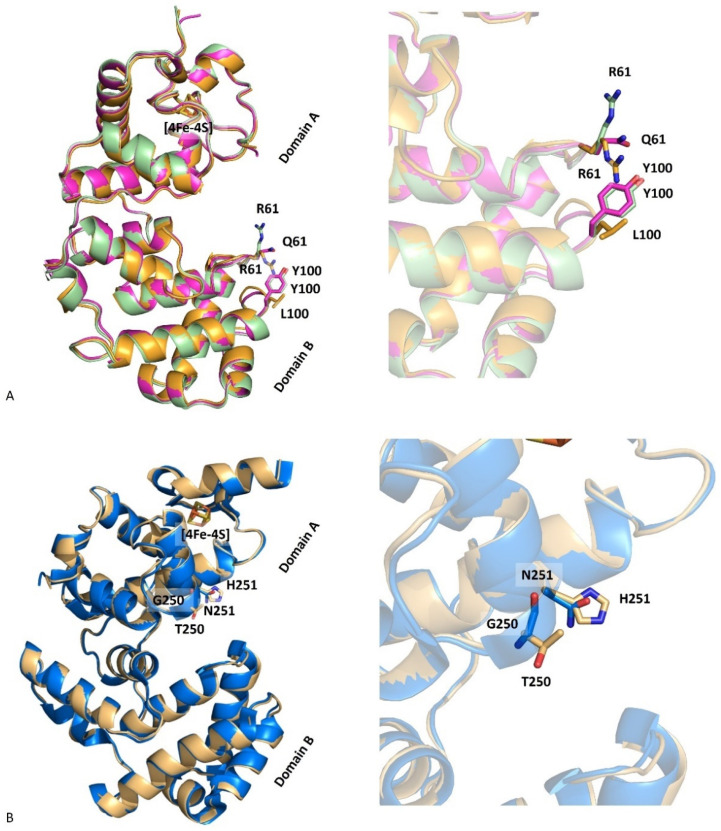
Superimposition of DrEndoIII1 native (cyan, 4UNF), R61Q (pink, 8A5F) and Y100L (orange, 8A5C) structures (**A**) and of DrEndoIII3 native (blue, 4UOB) and G250T/N251H (grey, 8A5G) (**B**).

A superimposition of the molecules from DrEndoIII1 R61Q and Y100L crystal structures showed a root-mean-square distance (*r.m.s.d.*) between Cα atoms of 0.50 Å. The structural superposition of DrEndoIII1 R61Q and Y100L with native DrEndoIII1 (PDB 4UNF) shows similar three-dimensional arrangements with overall Cα *r.m.s.d.s* of 0.39 Å and 0.52 Å, respectively (Figure 3A). The structures of DrEndoIII3 double mutant and native enzyme (PDB 4UOB) led to a Cα *r.m.s.d.* value of 0.52 Å (Figure 3B). Thus, our results show that the mutations did not induce any major structural changes in the overall structure of DrEndoIII1 and DrEndoIII3 and cannot explain the reduced activity of the DrEndoIII1 mutants or the failed “back to typical endonuclease III function” substitutions in DrEndoIII3. In the case of DrEndoIII1, this can be rationalized by altered charge and reduced substrate stabilization as suggested above. As for DrEndoIII3, the lack of activity was previously suggested to be caused by a poor steric fit of the substrate in the substrate binding pocket (demonstrated by MD simulations) [12]. As shown here, the GN to TH substitution neither altered the structure of this pocket, nor provided better fit of the substrate, therefore revealing no ability of the substrate to interact with the catalytic residues.

### 2.2. Biophysical Characterization

#### 2.2.1. Protein Thermal Stability

**Figure 4:**
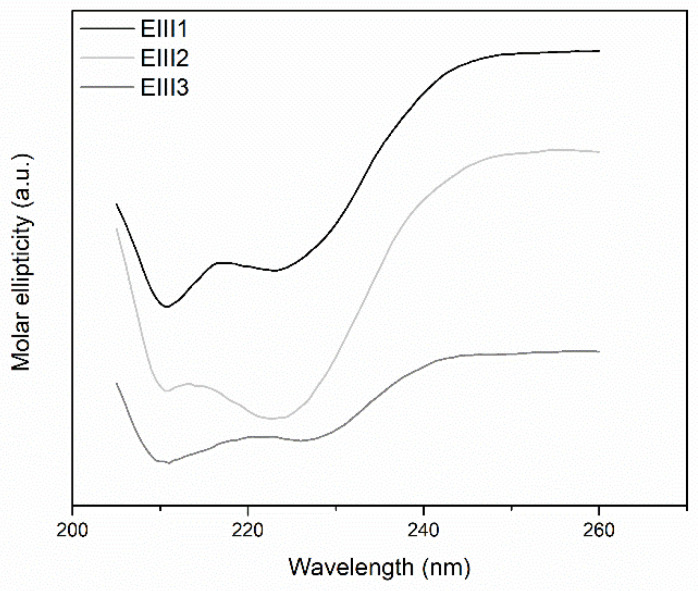
CD spectra of DrEndoIII 1, 2 and 3. The differences observed between the CD spectra of the wild-type enzymes are related to different concentrations used for each protein in order to obtain a spectrum (0.15 mg mL^−1^ for DrEndoIII1 and 2 and 0.05 mg mL^−1^ for DrEndoIII3).

Thermal stability of DrEndoIII1, 2 and 3 and the three mutants (R61Q, Y100L and G250T/N251H) was analyzed by CD spectroscopy in the 20 to 95 °C interval. The CD spectra demonstrate, as expected, a typical α-helical structure for the wild-type enzymes (Figure 4) and the mutants (Figure S2). The respective melting points (Tm) were determined by monitoring CD spectra during a stepwise increase of the temperature. The same Tm (around 60 °C) was observed for all enzymes, revealing that the mutations did not affect the stability of the proteins (Table S4).

#### 2.2.2. Electrochemical and RR Analysis

**Figure 5:**
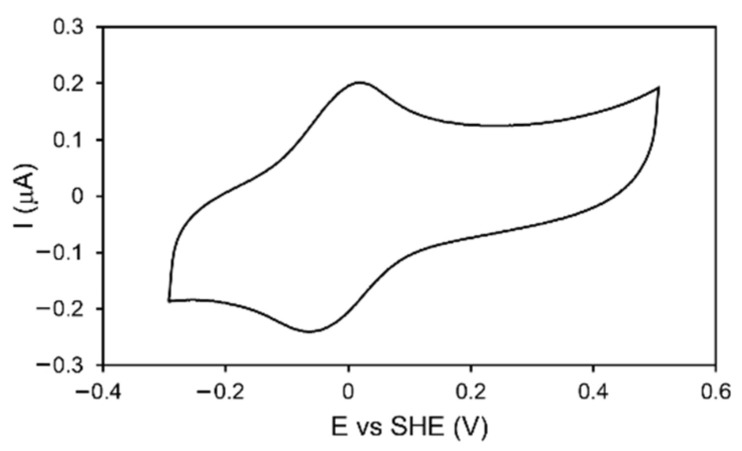
CV of DrEndoIII1 immobilized on Au electrodes coated with 11-mercaptoundecanoic acid (MUA) terminated SAM in 50 mM Tris-Hcl pH 8.5, 50 mM NaCl buffer. Scan rate: 50 mV/s.

The reduction potential of the [4Fe-4S] cluster in DrEndoIII1, DrEndoIII3 and the mutants was determined by cyclic voltammetry (CV). Both DrEndoIII1 and 3, immobilized on gold electrodes modified with MUA-terminated SAMs [16,17], like DrEndoIII2, possess a reduction potential of E° = − 20 mV +/− 10 mV (Figure 5 and Figure S3A–E). The electron transfer rate constants, derived from scan-rate-dependent measurements, are also comparable with those of DrEndoIII2 and other DNA glycosylases [15] (*ket*: DrEndoIII1—3.2 s^−1^; DrEndoIII3—2.1 s^−1^). The mutants showed comparable reduction potentials and electron transfer rate constants (*ket:* DrEndoIII1 R61Q—4.4 s^−1^; DrEndoIII1 Y100L—4.1 s^−1^; DrEndoIII3 G250T/N251H—2.9 s^−1^).

Furthermore, the structural features of the Fe-S clusters in DrEndoIII1, 2 and 3 and the three mutants (R61Q, Y100L and G250T/N251H) were probed by Resonance Raman (RR) spectroscopy. The RR spectra of all enzymes reveal vibrational fingerprint characteristics for all cysteinyl coordinated [4Fe-4S]^2+^ clusters (Figure S3F). As in the case of the previously studied DrEndoIII2, the bands at 339 cm^−1^ and 390 cm^−1^ are attributed to Fe-S stretching bridging modes, while the bands at 364 cm^−1^ and 372 cm^−1^ are ascribed to terminal Fe-S stretching [16].

Overall, the biophysical characterization demonstrates that the three DrEndoIII enzymes as well as the studied mutants possess the same thermal stability and redox properties as well as fingerprint characteristics of the [4Fe-4S] clusters. Thus, neither the different catalytic properties among the three wild-type enzymes, nor the altered biochemical properties observed of the mutants, can be rationalized by differences in thermodynamic or electronic properties of the cluster.

### 2.3. The Role of the FeS Cluster for DrEndoIII Enzymes

In order to probe the still-disputed role of the [4Fe-4S] cluster in EndoIII enzymes, we attempted to generate mutants of DrEndoIII2 by substituting the cysteines that coordinates the cluster Fe atom (C199, C206, C209, C215) (single mutants) with Ala or Ser. In this manner, we aimed at destabilizing the FeS cluster and evaluate how this affected the viability and activity of the protein.

First, Cys199 was substituted by an Ala (C199A) in DrEndoIII2, however no recombinant protein was obtained. An equivalent substitution has been reported for MutY, another HhH DNA glycosylase, which possesses a catalytic domain similar to EndoIII enzymes [18]. A replacement of Cys with Ser in MutY, resulted in low levels of overexpressed mutant [18]. Therefore, Cys to Ser substitutions were performed for all the cysteines in DrEndoIII2, however we were only able to obtain overexpression of the C199S mutant, nevertheless at a significantly lower yield than that of the wild-type enzyme. The resulting protein appeared less colored than the wild type at equivalent protein concentrations. It was also more temperature sensitive and unstable (precipitated at temperatures < 4 °C) and had to be kept at room temperature, at which it also precipitated after 16–48 h; it was not stabilized further by the addition of 10% glycerol.

Due to the stability issues, the protein was purified using only one HisTrap step (including buffer exchange to remove imidazole) and used immediately in the following experiments (apparent purity above 95%) (Figure S4).

**Figure 6:**
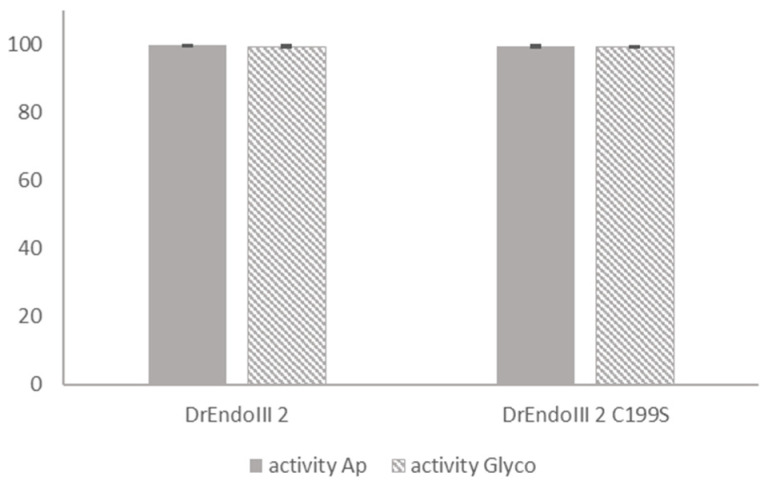
Comparative activity analysis between DrEndoIII2 and its mutant (DrEndoIII2 C199S). Processing of thymine glycol (Tg), in percentage, (plain bars) and Ab-site (striped bars) by DrEndoIII2 and DrEndoIII2 C199S. Histograms represent means and standard deviations of at least three independent experiments.

The activity of DrEndoIII2 C199S was measured using 5′-FAM labeled 35nt duplex oligos with a modified nucleobase (Tg) paired with a guanine in a protein:DNA ratio of 1:1 (75 nM) in an end point analysis and compared to the activity of DrEndoIII2 using the same conditions. The results showed that the activity of the mutant was comparable to that of DrEndoIII2, indicating that the activity of the protein was not compromised by the substitution of C199 to S (Figure 6 and Figure S1).

**Figure 7:**
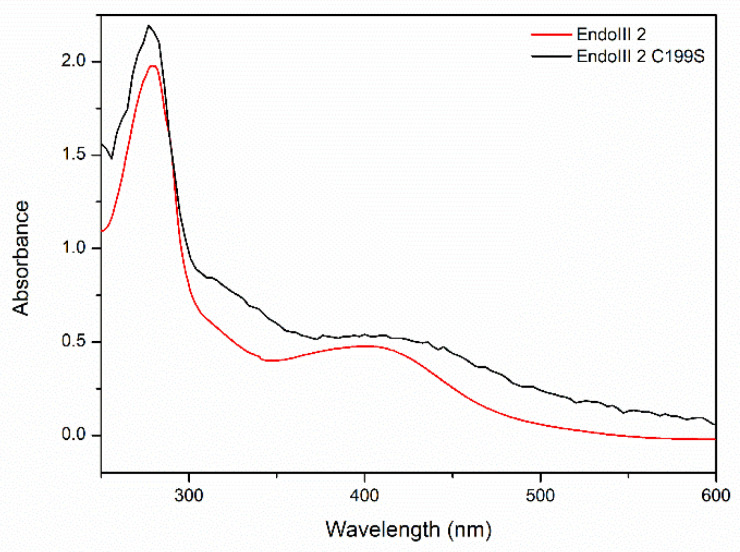
UV-vis spectra of DrEndIII2 and DrEndoIII2 C199S.

We further characterized the structural and redox properties of the [4Fe-4S] cluster in the DrEndoIII2 C199S mutant. The UV-vis spectra demonstrated small alterations in comparison with the wild-type enzyme, exhibiting lower absorbance of iron-sulfur clusters (around 410 nm), which we attribute to cluster degradation, which inevitably occurs over time (Figure 7). The CV experiments, show redox transition (E°) of 30 mV +/− 10 mV for DrEndoIII2 C199S, which is comparable with that of the wild type at the same pH [17] (Figure S5).

**Figure 8:**
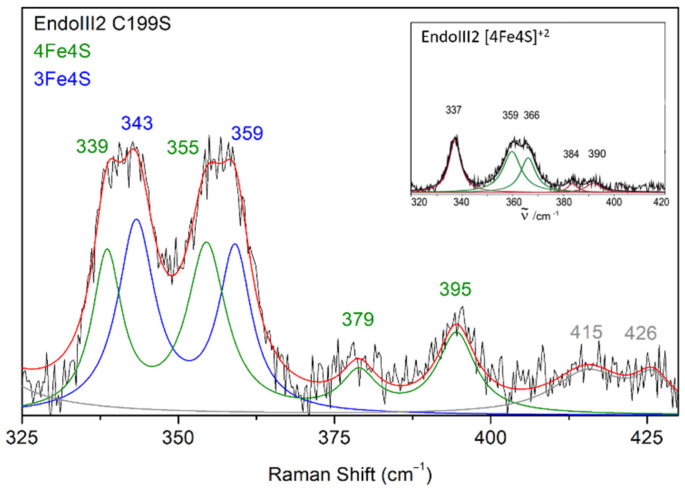
Experimental RR spectrum of DrEndoIII2 C199S in the resting state obtained using 413 nm excitation (black trace) and its component spectrum with designated vibrational modes that originate from 4Fe-4S (green trace) and 3Fe-4S clusters (blue trace). Inset RR spectrum of DrEndoIII2 native enzyme that possesses only 4Fe-4S cluster (adapted from [17] with permission from Elsevier).

Fine structural details on the cluster in DrEndoIII2 C199S, obtained by RR spectroscopy, reveal a presence of co-existing [3Fe-4S] and [4Fe-4S] cluster populations (Figure 8).

The bands at 339 and 355 cm^−1^ originate from the [4Fe-4S] and 343 and 359 cm^−1^ from the [3Fe-4S] cluster. RR spectra measured at different time points after protein purification, indicate a gradual degradation of the [4Fe-4S] into [3Fe-4S] cluster; the amount of the latter increases along 16 h from 74% to 85% (Table S5).

Site-directed mutagenesis has commonly been performed to obtain FeS proteins with non-cysteine ligands. Such alterations often result in a cluster with an alternative ligand, a modified cluster, or a variant with no cluster [18,20,21]. Here, the tentative substitution of four iron-coordinating cysteines in the FeS cluster of DrEndoIII2, allowed for expression and purification of one mutant (DrEndoIII2 C199S), which showed compromised stability. This has previously been reported in the case of the HiPIP from *Chromatium vinosum,* where only one unstable Cys-Ser variant of the [4Fe-4S] cluster could be isolated, which nevertheless degraded within a few hours due via formation of another cluster species [21]. For MutY it was also reported that Cys to Ser substitution severely affected the stability and expression [18]. However, in this case it was shown possible to refold the protein in vitro and obtain a fully folded protein without the [4Fe-4S] cluster. The, protein was not active, which was explained with the lack of ability to bind substrate DNA [18].

Our results demonstrated that apart from its unstable character, the C199S mutant possesses comparable activity and redox properties to that of the wild-type DrEndoIII2. This suggests that the protein activity may not be directly dependent on the redox properties of the FeS cluster. However, it may be indirectly dependent on the cluster by providing an appropriate scaffold for substrate binding, allowing removal of the damaged base by the catalytic residues.

## 3. Concluding Remarks

Understanding the mechanistic properties, individual particularities, and the role of [4Fe-4S] cluster in the three DrEndoIIIs present in *D. radiodurans* is a challenging task. We have addressed here several hypotheses, derived from the enzyme’s structural differences, which appeared to be candidates for rationalizing their distinct catalytic properties. We demonstrate that the Arg and the Tyr in the DNA interacting loops of DrEndoIII1 are important for the properties of this enzyme, and that the molecular determinants underlying the catalytic activity of DrEndoIII3 in *D. radiodurans* require additional experimental work. Moreover, an unstable Cys to Ser variant of DrEndoIII2 was obtained with comparable activity and redox properties to the wild-type enzyme. However, due to the unstable properties of the mutant, additional experiments are needed to probe if the cluster is needed directly through its redox properties and/or indirectly by providing appropriate substrate binding for catalysis to take place.

## 4. Materials and Methods

### 4.1. Construction of Mutants

The constructs containing the genes encoding the endonuclease III enzymes (DrEndoIII1, DrEndoIII2 and DrEndoIII3) from *D. radiodurans* were the same as described previously by Sarre et al. 2014 [22]. The mutants (DrEndoIII1 R61Q, DrEndoIII1 Y100L, DrEndoIII2 C199A, DrEndoIII2 C199S, DrEndoIII2 C206S, DrEndoIII2 C209S, DrEndoIII2 C215S, DrEndoIII3 G250T/N251H, DrEndoIII3 R139D) were generated using the oligonucleotides described in Table S1 and the QuikChange II site-directed mutagenesis kit according to the manufacture’s instruction (Agilent, Santa Clara, CA, USA).

### 4.2. Protein Expression and Purification

The DrEndonucleaseIII wild-type and mutant enzymes were produced in *E. coli* BL21(DE3) pLysS. Cells were grown overnight at 37 °C, 150 rpm in 10 mL PB (Power Broth from Molecular Dimensions), medium with 100 µg mL^−1^ ampicillin and 34 µg mL^−1^ chloramphenicol. Cultures (1 L) were grown after inoculation with overnight preculture, at 37 °C, 150 rpm until the OD_600_ reached 0.6–0.8. Expression was induced by adding 0.5 mM isopropyl β-D-1-thiogalactopyranoside (IPTG) at 18 °C, 180 rpm. Cells were harvested by centrifugation at 4 °C, 8671× *g* for 30 min, and resuspended in 20 mL 50 mM Tris-HCl pH 7.5, 150 mM NaCl for DrEndoIII1 and DrEndoIII2 or 50 mM Tris-HCl pH 8.5, 150 mM NaCl for DrEndoIII3. Cells were lysed in the presence of lysozyme, DNaseI, 1 mM MgCl_2_ and protease-inhibitor tablets (Roche, Basel, Switzerland), by four cycles of freeze-thaw. Lysates were then centrifuged at 4 °C, 39,191× *g* for 30 min.

The supernatants collected after cell lysis were purified at room temperature by ӒKTA FPLC (Cytiva, Marlborough, MA, USA) in a three-step process using a 1 mL HisTrap column followed by a 5 mL Heparin column and a 10/30 Superdex 75 column. The soluble fraction was applied to a Histrap column (Cytiva, Marlborough, MA, USA) previously equilibrated with buffer A (50 mM Tris-HCl pH 7.5 (or 8.5), 150 mM NaCl). Proteins were eluted between 150–250 mM imidazole, during a gradient of 0–100% buffer B (50 mM Tris-HCl pH 7.5 (or 8.5), 150 mM NaCl, 500 mM imidazole). Further, in order to remove the imidazole, buffer exchange with buffer A was performed in PD-10 columns (Cytiva, Marlborough, MA, USA).

To remove potentially bound DNA from the purified proteins, a Heparin column (Cytiva, Marlborough, MA, USA) was used as a second purification step. Prior to sample application, the column was equilibrated in buffer A, and the samples were eluted in a single peak at 150 mM NaCl, using a gradient of 0–100% buffer B (50 mM Tris-HCl pH 7.5 (or 8.5), 1 M NaCl). The peak fractions were concentrated and applied to a 10/30 Superdex 75 size exclusion column (Cytiva, Marlborough, MA, USA) equilibrated in buffer A, where a single peak was obtained. The presence of the [4Fe-4S] was confirmed by UV-vis spectroscopy and the purity of the fractions was analyzed by SDS-Page and estimated to be greater than 95%. All the enzymes were concentrated until 20 mg mL^−1^ and stored at −80 °C.

### 4.3. Crystallization and Cryo-Protection

Initial crystallization screenings were performed at 20 °C in a Mosquito nano-drop protein dispenser robot (SPT Labtech, Cambridge, UK) using the sitting drop vapor-diffusion method. The following commercial screens (Molecular Dimensions, Florida, FL, USA) were used: JCSG plus, Structure I and II, BCS, Natrix, Nucleix suite, Morpheus, AmSO_4_ suite, Grid Screens (Ammonium Sulfate). DrEndoIII1 R61Q and Y100L mutants crystals were obtained in several conditions but the most promising one was from the condition number 49 (Structure I and II) consisting of 0.1 M bicine pH 9.0, 30% (*w*/*v*) PEG 500 MME and 0.1 M sodium chloride. Following this crystallization hit, microliter scale optimization proceeded using the hanging drop vapor-diffusion method in XRL 24-well crystallization plates (Molecular Dimensions). Crystals of DrEndoIII1 mutants appeared after three days in drops consisting of 1 µL protein solution (at 10 mg mL^−1^) and 1 µL of reservoir solution against 500 µL reservoir solution in the well. DrEndoIII3 G250T/N251H crystals were obtained in condition number 6 from the AmSO_4_ suite screen, composed by 0.2 M ammonium formate and 2.2 M ammonium sulfate. Crystals of DrEndoIII3 double mutant appeared after seven days in drops consisting of 1 µL protein solution (at 15 mg mL^−1^) and 2 µL of reservoir solution. The DrEndoIII1 and DrEndoIII3 mutants crystals were cryo-protected using the reservoir solution supplemented with 15% (*v*/*v*) glycerol prior to flash-cooling in liquid nitrogen.

### 4.4. Data Collection and Processing

DrEndoIII1 and DrEndoIII3 mutants X-ray diffraction data were collected at 100 K at ALBA synchrotron (Barcelona, Spain) on the BL13-XALOC beamline equipped with a PILATUS 6M detector and an MD2M diffractometer [23]. XDS [24] was used for diffraction spots indexing, integration, scaling, and merging into the final amplitudes dataset. DrEndoIII1 and DrEndoIII3 mutants data were processed in space group C2. Data collection details and respective processing statistics are listed in Table S2.

### 4.5. Structure Determination and Refinement

Estimation of the DrEndoIII1 and DrEndoIII3 mutant’s unit cell contents was obtained with the Matthews Probability Calculator, which estimated one molecule in the asymmetric unit [25,26]. The DrEndoIII1 R61Q and DrEndoIII3 double mutant phase problems were solved by molecular replacement using MORDA [27] and the structure coordinates of *Deinococcus radiodurans* Endonuclease III1 (PDB 4UNF) [4], and *Deinococcus radiodurans* Endonuclease III3 (PDB 4UOB) [4], as search models, respectively. As the DrEndoIII1 Y100L was isomorphous with the DrEndoIII1 R61Q crystal, the monomer in the asymmetric unit was refined with PHENIX.REFINE [28,29,30] using an initial rigid-body refinement followed by refinement of atomic positions, isotropic atomic displacement parameters (a.d.p.s) and domains of translation, liberation, and screw (TLS) refinement of a.d.p.s which had been previously defined with the TLSMD server (http://skuld.bmsc.washington.edu/~tlsmd) (accessed on 23 November 2021) [31]. Cycles of iterative models inspection and edition against σA electron density maps with COOT [32] were alternated with model refinement. Although refinement included standard stereochemistry libraries [33] the inter-atomic distances involving iron-sulfur centers were refined without target restraints. Approximately 10% of reflections were randomly chosen for Rfree monitoring. Solvent water molecules were automatically assigned from σA difference maps peaks neighboring hydrogen bonding acceptors/donors within 2.45–3.40 Å distances. Other solvent molecules were identified through comparison of their shapes against electron density blobs, as well as by comparing their refined a.d.p.s with those of neighboring atoms. The stereochemistry of the refined structures was analyzed with MOLPROBITY [34]. Figures of structural models were prepared with PyMOL [35,36]. Refinement statistics are presented in Table S2. Structure factors and associated structure coordinates of DrEndoIII1 R61Q, DrEndoIII1 Y100L and DrEndoIII3 G250T/N251H were deposited in the Protein Data Bank (www.rcsb.org) (accessed on 15 June 2022) [37] with PDB codes 8A5F, 8A5C and 8A5G, respectively.

### 4.6. Activity Assays

Gel-based activity measurements were performed as described in Sarre et al. 2019 [12], using 5′-FAM labeled 35 nt dsDNA oligonucleotides (oligos) with a modified nucleobase in position 14 (Table S3). Oligos containing oxidized bases (Tg, U) were synthesized by Midland Certified Reagent Co. (Texas, TX, USA) and complementary strands were purchased from MWG Eurofins (Ebersberg, Germany).

### 4.7. EMSA

To test the binding affinity of DrEndoIII enzymes to DNA, two substrates were used. A 45mer 5′-FAM labeled DNA oligo containing a stable THF-abasic site (Table S1) annealed to a complementary strand with a G opposite the damaged site and an undamaged 45 mer labeled DNA oligo (Table S1) also annealed with a complementary strand. 100 nM of both substrates was mixed with increasing concentrations of DrEndoIII enzymes (0, 0.125, 0.5, 2 and 8 M) in 50 mM Tris pH 7.5 and 150 mM NaCl and incubated at 25 °C for 10 min.

The samples were then separated by electrophoresis on a 10% TBE native acrylamide gel at 4 °C during 90 min and the bands were visualized on a Fuji TLA-5100 scanner using a LPB filter (473 nm).

### 4.8. Circular Dichroism

Circular dichroism (CD) spectroscopy was used to analyze the secondary structure of native and mutated DrEndoIIIs enzymes in 50 mM Tris-HCl pH 7.5 (or 8.5), 50 mM NaCl. CD spectra were recorded on a Jasco J-815 CD spectrometer using a cell of 0.02 cm path length. Spectral accumulation was performed with a scanning rate at 50 nm min^−1^ over the wavelength range of 190–260 nm for far-UV CD measurements. Each spectra were obtained from an average of 10 scans. Protein concentration of 0.15 to 0.05 mg mL^−1^ was used, and the spectra were normalized with the buffer.

### 4.9. Electrochemistry

Cyclic Voltammetry (CV) experiments were performed in a three-electrode electrochemical cell arrangement composed of a Ag/AgCl (3 M, KCl) reference electrode (+0.21 V vs. SHE), a platinum wire as a counter electrode and a Au working electrode (BASi). The supporting electrolyte, 50 mM Tris-HCl pH 7.5 (or 8.5), 50 mM NaCl, was thoroughly purged with argon. Electrode potentials were controlled by a Princeton Applied Research 263A potentiostat (Oak Ridge, TN, USA). Scan rate dependence was performed in the range of 5–1000 mV/s, in the potential window from −0.3 to +0.5 V, and the ET rate constants were determined using Laviron method [38]. Gold electrodes were subjected to piranha solution for ~5 min, polished with 1, 0.3 and 0.05 µm alumina and cleaned by ultrasounds in water for 5 min. Electrodes were then rinsed and dried with compressed air and functionalized by immersion in a 1 mM solution of 11-mercaptoundecanoic acid (MUA) in ethanol to form a self-assembled monolayer (SAM). Prior to CV measurements, the SAM coated electrodes were incubated with a droplet of concentrated enzyme solution (0.4–0.75 mM) deposited on the electrode surface for ~20 min; after protein incubation the electrodes were thoroughly rinsed with buffer to remove the non-bound molecules.

### 4.10. Resonance Raman

Resonance Raman (RR) spectroscopic experiments were performed with 2 μL of 0.5 mM enzyme introduced into a liquid-nitrogen-cooled cryostat (Linkam, THMS 600, Tadworth, UK), mounted on a microscope stage, and cooled down to 77 K. Spectra from the frozen samples were collected in backscattering geometry by a confocal microscope coupled to a Raman spectrometer (Jobin Yvon U1000, Edison, NJ, USA) equipped with 1200 1/mm grating and a liquid nitrogen cooled CCD detector. The 413-nm line from a krypton ion laser (Coherent Innova 302, Santa Clara, CA, USA) was used as excitation source. Spectra were accumulated for 60 s with a laser power of 8 mW at the sample with the background scattering removed by subtraction of a polynomial function.

## Data Availability

Not applicable.

## Supplementary Materials

The following are available at: https://www.mdpi.com/article/10.3390/molecules27134270/s1. Table S1. Primers for mutagenesis, Table S2. X-ray data collection and refinement statistic, Table S3. Oligonucleotides used for probing DrEndoIII activity, Table S4. DrEndoIII native and mutant enzymes temperature melting point, Table S5. Determination in percentage the formation of [3Fe-4S] cluster formation with time, Figure S1, Activity assays (denaturing gels), Figure S2. CD spectra of mutants, Figure S3. CV and RR spectra of DrEndoIII wild-type and mutants, Figure S4. Chromatogram and SDSPAGE of purified DrEndoIII2 C199S mutant, Figure S5. CV of DrEndoIII2 C199S mutant.
