# Supplementary material for: Disentangling Unusual Catalytic Properties and the Role of the [4Fe-4S] Cluster of Three Endonuclease III from the Extremophile D. radiodurans
Source: Molecules. 2022 Jul 2;27(13):4270. doi: 10.3390/molecules27134270 (PMC9268735; doi:10.3390/molecules27134270)
Supplement: Supplementary file 1 [file molecules-27-04270-s001.zip › molecules-1796566-supplementary.pdf]

Supplementary tables

Table S1. Primers for mutagenesis.

Primers	Sequence
**EndoIII1 R61Q**	Fw ATCCTCTCGCAG**CAG**ACCACCCACGCCGAC<br>Rv GTCGGCGTGGGTGG**TCT**GCTGCGAGAGGAT
**EndoIII1 Y100L**	Fw CGCCGCAGCAAT**CTG**CCTGAGAGCAAGGCC<br>Rv GGCCTTGCTCTCAGG**CAG**ATTGCTGCGGCG
**EndoIII2 C199A**	Fw GCCGCCGCGT**CGC**CCACGCCCGCA<br>Rv TGCGGGCGTGG**GCG**ACGCGGCGGC
**EndoIII2 C199S**	Fw CCGCCGCGT**CAG**CCACGCCCG<br>Rv CGGGCGTGG**CTG**ACGCGGCGG
**EndoIII2 C206S**	Fw GCAAGCCCC**AGA**GCCCGTCGTGC<br>Rv GCACGACGGG**CTC**TGGGGCTTGC
**EndoIII2 C209S**	Fw CCA GTG CCC GTC **GAG** CGA ATT GGC GAG<br>Rv CTC GCC AAT TCG **CTC** GAC GGG CAC TGG
**EndoIII2 C215S**	Fw GAATTGGCGAGCTTC**AGT**CCGAAAGTAGGGG<br>Rv CCCCTACTTTCGGAC**TGA**AGCTCGCCAATTC
**EndoIII3 G250TN251H**	Fw ATGCCGGTGGAT**ACCCAC**ATGGAACGGGCG<br>Rv CGCCCGTTCCAT**GTGGGT**ATCCACCGGCAT
**EndoIII3 R139D**	Fw GCAGCAAAACAC**CGA**CCGGGTCGCGCAG<br>Rv CTGCGCGACCCG**GTC**GGTGTTTTGCTGC

Table S2. X-ray data collection and refinement statistics. Values in parentheses belong to the highest resolution shell.

	**EIII1 R61Q**	**EIII1 Y100L**	**EIII3 G250T/N251H**
**Data collection**			
Beamline	ALBA XALOC	ALBA XALOC	ALBA XALOC
Wavelength (Å)	0.97926	0.97926	0.97926
Space group	*C 1 2 1*	*C 1 2 1*	*C 1 2 1*
Unit cell parameters (Å, °)	a = 179.3, b = 38.0, c = 36.4, β= 91.0	a = 178.5, b = 37.8, c = 36.6, β= 90.6	a = 90.3, b = 38.8, c = 72.0, β= 99.7
Resolution (Å)	44.82 – 1.38 (1.47 – 1.38)	44.63 - 1.95 (2.05 – 1.95)	44.53 – 1.89 (1.99 – 1.89)
Nr. observations	146093 (22818)	56386 (9040)	65506 (9163)
Nr. unique reflections	50163 (8090)	17563 (2766)	19937 (3111)
Completeness (%)	98.9 (99.1)	97.1 (96.2)	99.1 (97.6)
Multiplicity	2.9 (2.8)	3.2 (3.3)	3.3 (2.9)
Mosaicity (°)	0.16	0.14	0.15
$CC_{1/2}$ (%)[a]	99.4 (46.2)	99.7 (49.7)	99.9 (49.8)
R_{sym} (%)[b]	5.8 (89.6)	4.9 (74.9)	4.2 (54.4)
R_{meas} (%)[c]	7.2 (83.9)	6.2 (91.4)	5.9 (71.2)
$R_{p.i.m.}$ (%)[d]	4.0 (31.3)	3.4 (31.1)	3.0 (28.3)
$\langle I/\sigma(I) \rangle$	8.85 (0.99)	9.88 (1.07)	13.71 (1.61)

Wilson B (Å^2)	31.6	56.7	39.1
V_M (Å^3 Da^{-1})	2.08	2.08	1.98
Estimated solvent content (%)	41.0	40.8	37.8
**Refinement**			
R_{work} (%)[e]	15.1	19.4	23.8
R_{free} (%)[e]	18.0	24.7	28.9
rmsd for bond lengths (Å)	0.005	0.008	0.008
rmsd for bond angles (°)	0.696	0.824	0.870
Structure <*a.d.p.*> (Å^2)	36.1	75.8	50.6
Ramachandran plot			
Residues in favored regions (%)	97.9	94.5	97.0
Residues in allowed regions (%)	2.1	5.5	3.0
Residues in disallowed regions (%)	0	0	0
PDB code	8A5F	8A5C	8A5G

[a] $CC_{1/2}$ = Percentage of correlation between intensities from random half-datasets (Karplus & Diederichs, 2012).

[b] R_{sym} = Σhkl Σi |Ii(hkl) - <I(hkl)>|/ Σhkl Σi Ii (hkl), where Ii(hkl) is the observed intensity and <I(hkl)> is the average intensity of multiple observations from symmetry-related reflection (Arndt et al., 1968).

[c] R_{meas} = Σhkl [N/(N(hkl) -1)]1/2 Σi |Ii(hkl) - <I(hkl) >|/Σhkl Σi Ii (hkl), where N(hkl) is the data multiplicity, Ii(hkl) is the observed intensity and <I(hkl)> is the average intensity of multiple observations from symmetry-related reflections. It is an indicator of the agreement between symmetry related observation (Diederichs & Karplus, 1997).

[d] $R_{p.i.m.}$ = Σhkl [1/(N(hkl) -1)]1/2 Σi |Ii(hkl) - <I(hkl) >|/Σhkl Σi Ii (hkl), where N(hkl) is the data multiplicity, Ii(hkl) is the observed intensity and <I(hkl)> is the average intensity of multiple observations from symmetry-related reflections. It is an indicator of the precision of the final merged and averaged data set (Weiss, 2001).

[e] R_{work} refers to the actual working data set used in refinement, while R_{free} refers to a cross validation set that is not directly used in refinement and is therefore free from refinement bias.

Table S3. Oligonucleotides used for probing DrEndoIII activity.

SUBSTRATE	SEQUENCE
**TG SUSBTRATE (6-FAM)**	TGTCAATAGCAAG(**ThyGly**)GGAGAAGTCAATCGTGAGTCT
**URACIL SUSBTRATE (6-FAM)**	TGTCAATAGCAAG**U**GGAGAAGTCAATCGTGAGTCT
**REVERSE G**	AGACTCACGATTGACTTCTCC**G**CTTGCTATTGACA
**REVERSE A**	AGACTCACGATTGACTTCTCC**A**CTTGCTATTGACA
**THF SUBSTRATE (6-FAM)**	AGCTACCATGCCTGCACGAA**X**TAAGCAATTCGTAATCATGGTCAT
**REVERSE G**	ATGACCATGATTACGAATTGCTTA**G**TTCGTGCAGGCATGGTAGCT
**FORWARD C**	AGCTACCATGCCTGCACGAA**C**TAAGCAATTCGTAATCATGGTCAT

Table S4. DrEndoIII native and mutated enzymes temperature melting point.

**Protein**	TM (°C)
EndoIII1	63
EndoIII2	62
EndoIII3	61
EndoIII1 R61Q	62.4
EndoIII1 Y100L	62.13
EndoIII3 G250T/N251H	60

Table S5. Determination in percentage through RR, of the amount of [3Fe-4S] and [4Fe-4S] present in solution in DrEndoIII2 C199S.

time (h)	[3Fe-4S]	[4Fe-4S]
0	74	26
5	79	21
16	85	15

Supplementary Figures

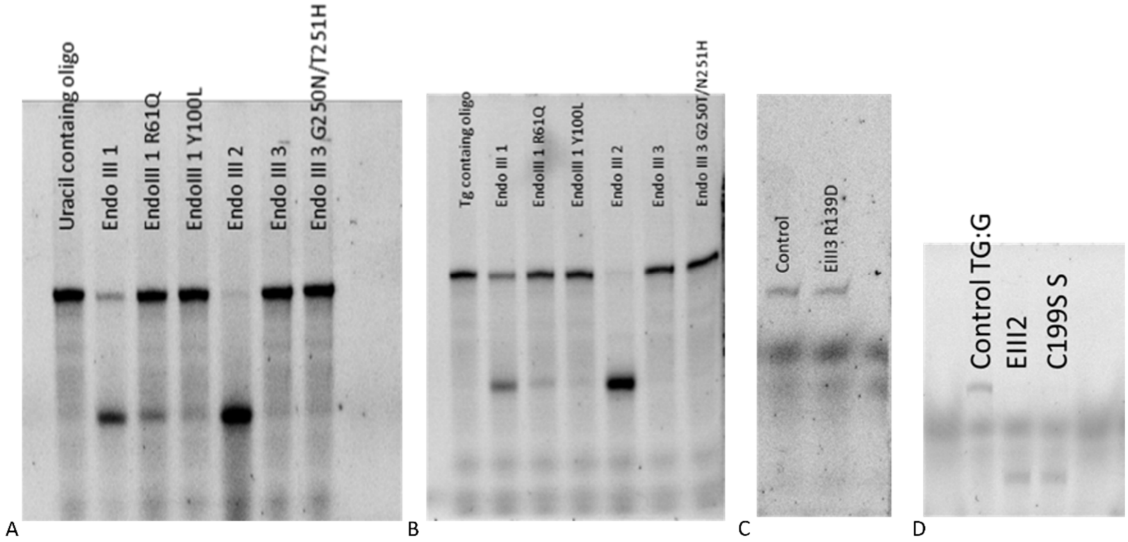

Figure S1. Denaturing gel electrophoresis analysis of the processing of Tg containing DNA by DrEndoIII1 and DrEndoIII2 and DrEndoIII3 (wild-type and mutants). AP-lyase activity (A), DNA glycosylase activity (B), activity of DrEndoIII3 R139D (C) and activity of DrEndoIII2 C199S (D).

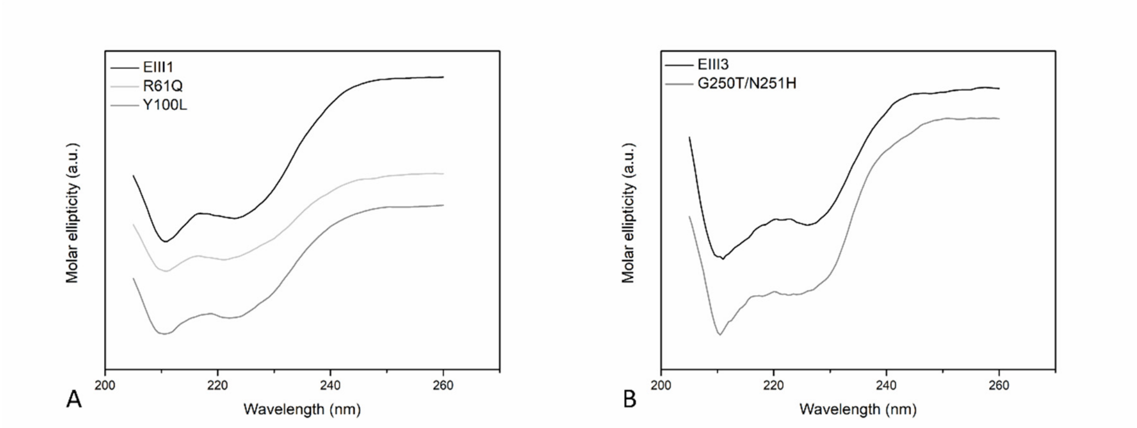

Figure S2. CD spectra of DrEndoIII1, DrEndoIII1 R61Q and Y100L (A), and DrEndoIII3 and DrEndoIII3 G250T/N251H (B).

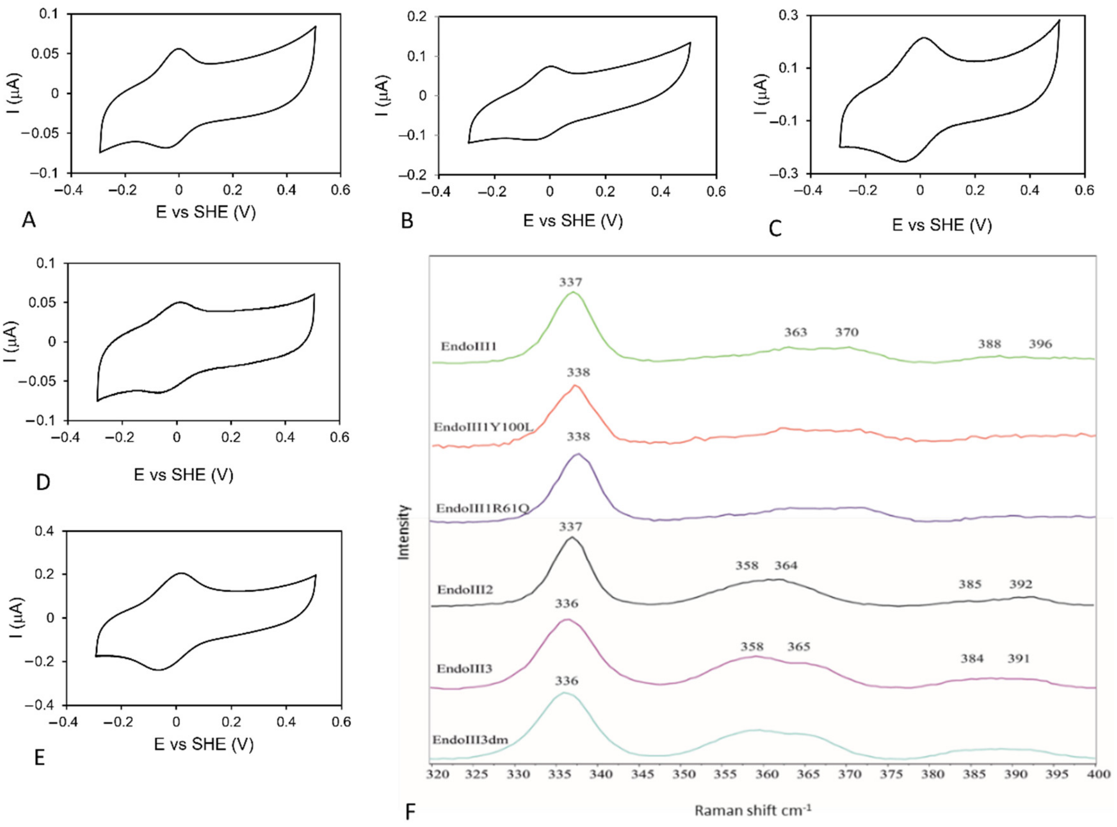

Figure S3. CV of DrEndoIII1 R61Q (A) and Y100L (B); DrEndoIII3 (C) and DrEndoIII3G250T/N251H (D); DrEndoIII2 (E), on Au electrodes coated with 11-mercaptoundecanoic acid (MUA) in 50 mM Tris-Hcl pH 8.5, 50 mM NaCl buffer. Scan rate: 50 mV/s. F) RR spectra DrEndoIII1, DrEndoIII1R61Q and DrEndoIII1Y100L; DrEndoIII2; DrEndoIII3 and DrEndoIII3G250T/N251H in the resting state.

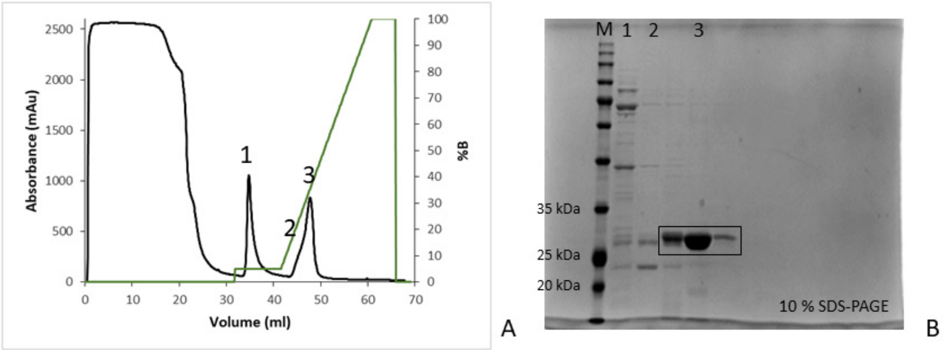

Figure S4. Chromatogram of HisTrap purification of DrEndoIII2 C199S (A) and SDSPAGE analysis of DrEndoIII2 C199S purification from different peaks (1, 2 and 3) highlighted in the chromatogram (B) (M- NZYColour protein marker II from NZYTech).

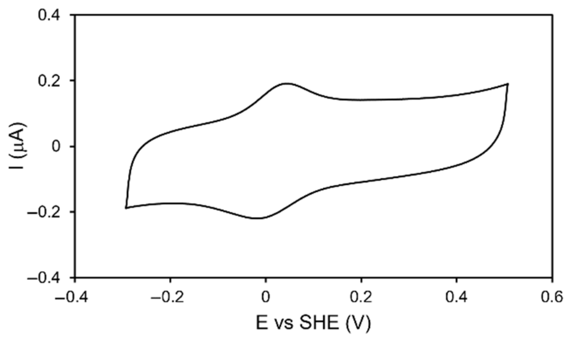

Figure S5. CV DrEndoIII2 C199S immobilized on Au electrodes coated with 11-mercaptoundecanoic acid (MUA) in 50 mM Tris-Hcl pH 7.5 (B), 50 mM NaCl buffer terminated SAM. V_{scan} 50 mV/s.
